# Demand generation activities and modern contraceptive use in urban areas of four countries: a longitudinal evaluation

**DOI:** 10.9745/GHSP-D-14-00109

**Published:** 2014-12-02

**Authors:** Ilene S Speizer, Meghan Corroon, Lisa Calhoun, Peter Lance, Livia Montana, Priya Nanda, David Guilkey

**Affiliations:** a The University of North Carolina at Chapel Hill, Gillings School of Global Public Health, Department of Maternal and Child Health, Chapel Hill, NC, USA; b The University of North Carolina at Chapel Hill, Carolina Population Center, Chapel Hill, NC, USA; c Harvard University Center for Population and Development Studies, Cambridge, MA, USA; d International Center for Research on Women, Asia Regional Office, Delhi, India; e The University of North Carolina at Chapel Hill, Department of Economics, Chapel Hill, NC, USA

## Abstract

Family planning is crucial for preventing unintended pregnancies and for improving maternal and child health and well-being. In urban areas where there are large inequities in family planning use, particularly among the urban poor, programs are needed to increase access to and use of contraception among those most in need. This paper presents the midterm evaluation findings of the Urban Reproductive Health Initiative (Urban RH Initiative) programs, funded by the Bill & Melinda Gates Foundation, that are being implemented in 4 countries: India (Uttar Pradesh), Kenya, Nigeria, and Senegal. Between 2010 and 2013, the Measurement, Learning & Evaluation (MLE) project collected baseline and 2-year longitudinal follow-up data from women in target study cities to examine the role of demand generation activities undertaken as part of the Urban RH Initiative programs. Evaluation results demonstrate that, in each country where it was measured, outreach by community health or family planning workers as well as local radio programs were significantly associated with increased use of modern contraceptive methods. In addition, in India and Nigeria, television programs had a significant effect on modern contraceptive use, and in Kenya and Nigeria, the program slogans and materials that were blanketed across the cities (eg, leaflets/brochures distributed at health clinics and the program logo placed on all forms of materials, from market umbrellas to health facility signs and television programs) were also significantly associated with modern method use. Our results show that targeted, multilevel demand generation activities can make an important contribution to increasing modern contraceptive use in urban areas and could impact Millennium Development Goals for improved maternal and child health and access to reproductive health for all.

## Abstract (precis)

Demand generation activities that were significantly associated with increased use of modern contraception in India (Uttar Pradesh), Kenya, Nigeria, and Senegal included: (1) community outreach activities, such as home visits and group discussions about family planning; (2) local radio programs; and (3) branded slogans and print materials circulated widely across the city. Television programming was also significant in India and Nigeria. Exposure to more activities may increase women's likelihood of using contraception.

## INTRODUCTION

Family planning helps save lives and can improve human welfare substantially. It prevents unintended pregnancies that may lead to abortion, limits the closely spaced and higher-order births most likely to lead to maternal death, and reduces pregnancies that might result in infants being born with HIV infection.^1^^–^^5^ Family planning can lower fertility rates even in the poorest countries, making it more feasible to achieve long-term national goals related to universal education, poverty reduction, and improved environmental conditions.^1^^,^^2^

Because of these manifold benefits, family planning is central to reaching the Millennium Development Goals (MDGs), specifically MDG Target 5.B, which is to attain universal access to reproductive health by 2015. This goal will continue beyond the MDG 2015 deadlines; in 2012, the global community launched the FP2020 initiative to reach 120 million new contraceptive users in developing countries by 2020.^3^^,^^6^ Countries that have made commitments under the FP2020 initiative are currently designing country-level strategies to increase access to and use of family planning.

Strong evaluation evidence is needed to inform these country-level family planning strategies. To date, much of the evidence is focused on short-term program outcomes (eg, with 6–18 month follow-up) and on geographically small programs or programs targeting specific groups.^7^^,^^8^ Some notable exceptions are the Matlab and Navrongo demographic surveillance sites that support longitudinal assessment of long-term impact,^9^^–^^11^ but these studies are based in rural settings. Rigorous, longitudinal evaluation evidence of family planning programs in urban settings is lacking.

As the world becomes more urbanized, cities—and slums within cities—are growing rapidly in high-fertility countries, resulting in an increased need to provide family planning and health services in urban settings.^12^ While urban residents tend to have better access to and use of health services and better health outcomes, this “urban advantage” does not emerge across all urban residents.^13^^,^^14^ In particular, the urban poor tend to have larger family sizes, have less knowledge of and access to family planning methods and services, and are less likely to use contraception than wealthier urban residents.^15^^,^^16^ For example, in Uttar Pradesh, India, Speizer and colleagues^15^ showed that the urban poor were less likely than their wealthier counterparts to use family planning; among those who were using it, the poor were more likely than the wealthy to use sterilization. This suggests that the urban poor use spacing methods less and have less choice than wealthier urban residents. This disadvantage among the urban poor has been shown in multiple sub-Saharan African settings as well.^16^ Furthermore, in some cases, the urban poor have family planning and fertility indicators that are as modest as or are worse than their rural counterparts.^16^

The urban poor are less likely to use family planning than their wealthier counterparts.

To address the need for family planning in rapidly urbanizing areas, particularly among the urban poor, in 2009 the Bill & Melinda Gates Foundation launched the Urban Reproductive Health Initiative (Urban RH Initiative) in 4 countries: India (Uttar Pradesh), Kenya, Nigeria, and Senegal. The underlying goal of the Urban RH Initiative is to increase modern contraceptive use by 20 percentage points in targeted urban areas, particularly among the urban poor. Each country program aims to meet the following 5 key objectives related to the supply, demand, and advocacy environments:

- Develop cost-effective interventions for integrating quality family planning with maternal and newborn health, HIV and AIDS, postpartum, and postabortion care programs (supply)
- Improve the quality of family planning services for the urban poor, with emphasis on high-volume clinical settings (supply)
- Test novel public-private partnerships and innovative private-sector approaches to increase access to and use of family planning by the urban poor (supply)
- Develop interventions for creating demand for and sustaining use of family planning among marginalized urban populations (demand)
- Increase funding and financial mechanisms and promote a supportive policy environment for ensuring access to family planning supplies and services for the urban poor (advocacy)

The locally designed programs of the Urban RH Initiative are being evaluated independently by the Measurement, Learning & Evaluation (MLE) project, which is led by the University of North Carolina at Chapel Hill Carolina Population Center in collaboration with the International Center for Research on Women. Findings from the MLE project will inform future family planning and reproductive health programs in the target countries and globally.

This article provides a summary of the Urban RH Initiative country programs, focusing on the demand creation activities, along with midterm (2-year) longitudinal evaluation results of the effect of the demand creation activities on modern contraceptive use.

## URBAN RH INITIATIVE INTERVENTIONS

In each of the 4 countries, a consortium of local and international organizations have engaged together to increase modern contraceptive method use in target urban sites over the 4 to 5-year project period. Table 1 presents a summary of key details about the country programs, including the initial intervention cities and the delayed intervention cities where the programs began after midterm (ie, start date about 2 years later). Country-level programmatic strategies to meet the above supply, demand, and advocacy objectives were informed by baseline quantitative and qualitative data from each country (and city); this led to use of various media forms and messages and service delivery strategies designed specifically for the different contexts.

**Table 1.** Summary of Urban Reproductive Health Initiative Country Programs

**Country**	**Project Name, Lead, and Website**	**Initial Intervention Cities**	**Delayed Intervention Cities**	**Key Programmatic Strategies at Launch**
India (Uttar Pradesh)	Urban Health Initiative (UHI), FHI 360, http://uhi-india.org/	Agra Aligarh Allahabad Gorakhpur	Moradabad Varanasi	**Demand Generation** Interpersonal communication: home visits by peer educators to provide women and men information, counseling, and referral; focus on LAPMs for pregnant women Mid-media: street plays, road shows, magic shows (low exposure) Mass media: radio and television with targeted messages **Supply Side Activities** Postpartum service integration: targeted FP information, counseling, and promotion during pregnancy and postpartum; ensure supplies and provider competencies to offer LAPMs Postabortion service integration: provide FP counseling and services during postabortion care Expand service delivery and quality Expand method choice Improve technical and client-provider interaction skills of providers Public-private partnerships Partnerships with Janani and other high-volume private facilities Strengthen routine and fixed day services for poor from slum communities Social marketing of condoms and pills **Advocacy** Focus on policy, advocacy, scale
Kenya	Tupange (“Let's Plan”), Jhpiego, www.tupange.or.ke/	Nairobi Kisumu Mombasa	Kakamega Machakos	**Demand Generation** Generate demand by addressing social norms and barriers that inhibit FP use Community mobilization Wide distribution of print project materials Local and mass media, including radio and television shows targeted to urban poor and young audiences **Supply Side Activities** Improve quality and accessibility of FP services through integration of services. Focus on facilities: close to slum/informal settlements; with high-volume attendance; and with high usage from slum/informal settlements Ensure contraceptive security throughout the life of the project and beyond by addressing poor forecasting and developing electronic stock-out reporting system Engage formal and informal private sector: work with selected private nurses and clinical officers to offer high-quality and low-cost comprehensive FP services **Advocacy** Advocacy for improved policy environment Capacity building and sustainability: build capacity of local implementing partners, policy makers, private and public-sector providers to respond to FP/RH goals and needs
Senegal	L'Initiative Sénégalaise de Santé Urbaine (ISSU) (“Senegal Urban Reproductive Health Initiative”), IntraHealth International, www.facebook.com/sante.urbaine	Dakar Guédiawaye Pikine Mbao	Mbour Kaolack (outside the region of Dakar)	**Demand Generation** Outreach workers identifying FP needs Theater to promote discussion on a topic Small group discussions led by midwives with users to discuss FP-related topics Engagement of religious and community leaders to participate in and lead FP discussions Radio and television using public, private, and community-level stations **Supply Side Activities** Integration of FP into MCH services including postpartum and postabortion care Train providers to use cost-effective and evidence-based service delivery systematic screening tool to identify unmet FP needs Expand availability and quality of long-acting FP services in health facilities Train providers; ensure stock reliably available Integrate trained midwives into facilities to increase access to and availability of FP on a regular basis Outreach through mobile clinics targeting poor areas Social franchise strategies to increase access through the private sector Use Blue Star to increase access to FP in existing private-sector services **Advocacy** Advocacy to create a favorable policy environment
Nigeria	Nigerian Urban Reproductive Health Initiative (NURHI), Johns Hopkins Center for Communication Programs, www.nurhi.org/	Abuja Ibadan Ilorin Kaduna	Benin City Zaria	**Demand Generation** Social mobilization: interpersonal communication activities to encourage discussion and reduce barriers of miscommunication and social stigma to normalize FP, undertaken in various settings including markets, special events; spread of branded items in numerous community settings Media: radio and television at the state and local levels; use local-language slogans for specific city radio programs; radio magazine entertainment-education program **Supply Side Activities** Improve quality and integrate high-volume facilities: train providers, ensure stock, improve facility environment including quality standards Test novel public-private partnerships: Family Planning Providers Network trains, markets, and supplies providers with what they need to provide appropriate FP services and networks the providers together Clinical services: performance improvement to ensure that clinical providers offer full menu of methods with quality counseling and integrated services Patent Medicine Store/pharmacist: provide information, basic counseling, and non-clinical FP methods as first-line providers **Advocacy** Advocacy to promote FP discussions in public forum and to encourage acceptance at all levels

Abbreviations: FP, family planning; LAPMs, long-acting and permanent methods; MCH, maternal and child health.

In **India**, the Urban Health Initiative (UHI) implemented the following demand creation interventions:

- Interpersonal communication activities, including home visits by peer educators (particularly to homes in slum communities) to provide family planning information, counseling, and referral to all women and men as well as to promote long-acting and permanent methods to women who had recently become pregnant
- Radio and television programs with targeted family planning messages to promote women's control over family planning, men's adoption of sterilization for a happy family, and the importance of long-acting and permanent method use after childbirth
- Some mid-media activities, including street plays, road shows, and magic shows; these activities are not assessed in this article as they were undertaken predominately in one city with low overall exposure

The demand creation activities of the **Kenya** Urban RH Initiative (Tupange) focused on addressing social norms and barriers that inhibit family planning use through:

- Community mobilization activities including distribution of consortium-developed print materials, such as posters, leaflets, and comic books
- Mass media activities using focused radio and television shows that targeted the young and poor. For example, one of the Kenya project partners developed the *Jongo Love* program, a 24-episode radio drama series set in a fictional urban slum in Kenya, called Jongo, featuring the complex lives and relationships of residents and relating these to the reproductive choices they make. This series aired in Kenya just before implementation of the midterm survey.

In **Senegal**, the in-country consortium, l'Initiative Sénégalaise de Santé Urbaine (ISSU), implemented numerous types of demand creation activities targeting women and men, such as:

- Augmentation of a cadre of community outreach workers who identified family planning needs and counseled and referred women to family planning services
- Small group discussions on family planning-related topics led by project-engaged midwives
- Engaging and training religious and community leaders to become family planning champions and to lead family planning discussions in their local communities and at religious events
- Community theater to promote discussions on family planning topics within the community and between the community and religious leaders
- Radio and television programs aired on local public and private stations

The **Nigerian** Urban RH Initiative (NURHI), because it is based on the theory that creating demand for family planning will drive supply and consequently lead to long-term sustainability of program activities, developed numerous demand generation activities that together are thought to influence behavior change. These activities include:

- Social mobilization through interpersonal communication activities that encourage discussion about family planning and reduce barriers, myths, miscommunication, and social stigma against family planning. These activities are undertaken at numerous venues such as markets, association meetings, and special events including naming ceremonies, freedom ceremonies, graduation events, Christmas/Eid celebrations, and weddings.
- Mass media with a particular focus on local and state-level radio programs that promote local-language family planning slogans and messages, radio magazine entertainment-education programs (radio shows with various magazine elements, such as listener interviews and “ask the expert” segments), and television spots that promote the program's family planning messages and slogans
- Program brand/slogans/logos were used across program activities (not just on the radio and television) and were also spread to the community through badges worn by health care providers, umbrellas exhibited at the market, posters/displays in health facilities, and t-shirts, bracelets, and other items that were distributed extensively

The demand generation activities across the countries had 3 main themes: (1) fostering dialogue about family planning; (2) increasing social approval for family planning; and (3) improving knowledge and perceptions of family planning methods (Box). Not all countries promoted all the messages included in the box, but most countries promoted messages from each of the 3 main themes.

**BOX. Illustrative Themes of Family Planning Messages in Urban RH Initiative Programs**

**Foster dialogue about family planning at numerous venues**

- Model fertility and family planning discussions between couples (television, radio shows, posters)
- Model and promote family planning discussions with providers (posters, buttons, pamphlets)
- Train religious leaders to discuss family planning in groups and one-on-one (especially with men)
- Encourage young people to talk to providers and peers about family planning (radio dramas and programs)
- Promote wider societal discussion of family planning (mass events, radio, television)

**Increase social approval for family planning**

- Model healthy and happy families (radio dramas, television, posters)
- Promote role of family planning in responsible parenting (radio dramas, television, posters)
- Promote family planning for healthy timing and spacing of pregnancies (outreach events, radio, television)
- Develop and focus on one's life goals and family planning's role in achieving those goals (radio drama, comic book, outreach)
- Recognize and address reproductive choices of young people, poor people, and women (radio, television, outreach)

**Improve knowledge and perceptions of family planning methods**

- Address potential contraceptive side effects, myths, and misconceptions (leaflets, booklets, radio dramas, discussion groups)
- Promote postpartum family planning use and adoption of long-acting methods (radio talk shows, facility health talks)
- Increase knowledge of method choice and sources of methods (leaflets, radio, television, outreach)

## DATA AND VARIABLES

In each country, the MLE study design involves collecting longitudinal data from a representative sample of women prior to program implementation (baseline), at midterm (2 years after baseline), and at endline (4 years after baseline). In India, Kenya, and Nigeria, the baseline and midterm surveys were conducted in 2010 and 2012, respectively, and the endline survey will occur in 2014. In Senegal, each survey round occurred/will occur 1 year later (ie, 2011, 2013, and 2015). This article presents the baseline and midterm longitudinal evaluation results of the demand creation activities.

At **baseline** in each country, the MLE project used a 2-stage sampling design to obtain a representative sample of women from each of the initial and delayed intervention cities. In the first stage, for the 3 African countries, representative samples of primary sampling units (PSUs) in project cities were selected based on a sampling frame from the most recent census. In India, where the most recent census was nearly 10 years old, the MLE team developed a sample frame based on geographic location of residence designed to ensure adequate representation of the urban poor and selected a representative sample of PSUs for each study city. (Details of the geographic-based sampling approach used in India are provided elsewhere.^17^) For details of sampling and stratification of PSUs in each country, see the study reports.^18^^–^^21^ At the second stage of selection, following listing and mapping of each PSU, a systematic random sample of households was selected for interview.

Within selected households, all eligible women were interviewed at baseline. All women ages 15–49 years at the time of survey were eligible; the women had to be in union in India but not in the 3 African countries. Representative samples from each city were obtained (Table 2). Descriptions of the baseline samples can be found in the baseline country-level reports.^18^^–^^21^ All data collection activities were approved by the Institutional Review Board at the University of North Carolina at Chapel Hill and by the respective in-country ethics review boards.

**Table 2.** Number of Women Interviewed in Baseline and Midterm Surveys, by Country

**Country**	**Baseline, No.**	**Midterm, No. (% of Eligible Subsample Interviewed)**	**Samples for Longitudinal Analysis**
India (Uttar Pradesh)	17,643	5,790 (85.8%)	4,029 women interviewed at both baseline and midterm and who, at baseline, were in union, had not been sterilized, and had not had a hysterectomy
Kenya	8,932	3,207 (56.1%)	3,205 women interviewed at both baseline and midterm, regardless of their marital status at either time period, with non-missing data
Nigeria	16,144	4,331 (64.6%)	4,303 women interviewed at both baseline and midterm, regardless of their marital status at either time period, with non-missing data
Senegal	9,614	2,744 (80.7%)	1,538 women interviewed at both baseline and midterm and who were in union at baseline

Source: Measurement, Learning & Evaluation project baseline^18^^–^^21^ and midterm^22^^–^^25^ surveys of the Urban RH Initiative country programs.

At **midterm**, we tracked all women but interviewed only a subsample of them (Table 2).^22^^–^^25^ The donor reduced the size and scope of the midterm survey to obtain rapid results that the programs could use to implement midcourse corrections. Thus, the midterm evaluation focused on the demand generation activities because program activities to improve quality of and access to family planning services had not yet been implemented by midterm at wide enough scale to influence outcomes. These supply side measures will be included as part of the endline evaluation in 2014/2015. In collaboration with each country consortium, the MLE team designed a midterm survey that captured the full extent of demand generation activities being undertaken in each country, including information about women's exposure to mass media, mid-media, and interpersonal communication activities.

For copies of the baseline and midterm questionnaires, see the MLE project website at: www.urbanreproductivehealth.org/.

The primary outcome of interest in this analysis is modern contraceptive method use in each study country. Modern methods were defined as female or male sterilization, intrauterine devices (IUDs), implants, injectables, oral contraceptive pills, emergency contraception, condoms, the Lactational Amenorrhea Method, and other modern methods including spermicides, diaphragms, and the dermal patch, as applicable in each country.

For the longitudinal analysis presented in this article, we used slightly different study samples based on country-specific considerations (Table 2). For example, in Senegal, the analysis sample excluded women who were not in union at baseline because there is little non-marital sexual activity in the Senegalese context.

This article includes descriptive characteristics of the women interviewed at both time periods for each study country. The main demographic variables included in all multivariate models were age group, education, wealth status, religion, and, for Kenya and Nigeria only, marital status. Wealth group was created within country across cities based on household assets at each time point using principal components methods as done in the Demographic and Health Surveys.^26^ Thus, the wealth measures presented are based on the urban sample and provide a representation of which women were the poorest (and richest) in the urban areas under study.

The key exposure variables were country-specific variables related to specific program activities encompassing community activities, radio and television programming, print materials, and engagement of religious leaders.

All descriptive analyses are weighted using country-specific weights. These weights adjust for uneven probabilities of selection and non-response.

Estimation of multivariate models of the impact of women's recall of program exposure on modern contraceptive use by country used samples within which each woman had two observations—one from baseline and one from midterm. Estimation involved random effects logistic regression of an indicator for each woman's modern contraceptive use as of the panel (baseline or midterm) taking into account her background characteristics and program exposure as of that panel. Program exposure was, by definition, zero at baseline in nearly all cases (with the exception of one program component in India that had already been started by baseline), while for midterm the woman's actual empirically observed program exposure was used. Estimation thus exploited two panels of longitudinal data. Random effects were used to control for the correlation of each woman's observations between baseline and midterm.

The longitudinal data and estimation design has several advantages. First, it simply admits more variation in the key variables allowing for generally more precise estimation of the parameters of the model. Second, the longitudinal design also permits examination of the possible endogeneity (sometimes referred to as “confounding”) of the program exposure variables. Endogeneity involves possible correlation between interviewee recall of program exposure and some other unobserved determinants of modern contraceptive use that were not measured in the surveys, with the potential result that estimates of the impact of recall on modern contraceptive use will be biased. There are many potential unobserved determinants that might introduce such correlation (eg, cultural norms, preferences), but let us focus on health motivation as an example. If more motivated individuals are more likely to recall program exposure and to use contraception, then the intervening but unobserved motivation variable can lead to a misleading positive relationship between recall of program exposure and contraceptive use. Fortunately, one type of test of endogeneity can be exploited in the longitudinal data setting. The test involves comparison of results from an efficient estimator (ie, lower sample-to-sample variation in estimates) that does not correct for possible endogeneity of program exposure (random effects is such an estimator) with a less efficient estimator that does correct for the potential endogeneity of program exposure (fixed effects is the classic choice in the longitudinal data setting). Put simply, if the estimates yielded by the two models are similar, this suggests endogoneity is not playing a major role and any observed impact on contraceptive use is likely due to women's recall of program exposure.

The study’s longitudinal design has several advantages, namely that it can correct for possible endogeneity (or confounding) of the program exposure variables.

## RESULTS

### Baseline Demographic Characteristics

Table 3 presents the baseline demographic characteristics of the matched analysis sample by country. In Senegal and Uttar Pradesh, India, where the samples were of women in union, the analysis sample was older with fewer women in the 15–19 and 20–24 age groups. The samples in Kenya and Nigeria were somewhat more evenly distributed across the age groups.

**Table 3.** Baseline Demographic Characteristics of the Matched Baseline–Midterm Analysis Samples,^a^ by Country (%)

**Characteristic**	**India (UP) (N = 4,029)**	**Kenya (N = 3,205)**	**Nigeria (N = 4,303)**	**Senegal (N = 1,538)**
Age group				
15–19	3.48	9.42	16.17	3.99
20–24	18.75	25.15	15.21	14.42
25–29	24.28	23.64	18.65	18.49
30–34	21.84	15.97	16.52	19.92
35–39	15.73	12.33	14.39	18.38
40+	15.93	13.51	19.06	24.80
Education				
None/Quaranic	26.95	7.13	10.13	42.93
Primary	8.70	36.73	13.78	39.64
Secondary	36.99	39.80	48.49	15.67
Higher	27.35	16.34	26.70	1.77
Missing	0.00	0.00	0.89	0.00
Wealth group				
Poorest	22.50	16.76	14.49	15.49
Poor	21.44	19.66	17.92	22.53
Middle	18.90	21.26	19.95	26.43
Rich	19.11	21.67	22.41	19.28
Richest	18.05	20.64	25.22	16.27
Religion				
Hindi	80.98			
Catholic		21.96	5.05	
Protestant		63.86	42.01	
Muslim	19.01^b^	12.04	52.23	94.11
Other^c^				5.89
No religion		2.08	0.05	
Missing		0.06	0.66	
Marital status				
Never married	NA	25.71	30.11	NA
In union	NA	63.37	65.83	NA
Separated/divorced/widowed	NA	10.91	4.07	NA

Abbreviation: NA, not applicable (sample includes only women in union); UP, Uttar Pradesh.

a The matched analysis sample comprised, in India, women in union and not sterilized at baseline; in Kenya and Nigeria, all women; and in Senegal, women in union at baseline.

b Includes “other” category.

c Includes Christian and “other” categories.

Women in Senegal had the least formal education; 43% had no education or only Quaranic education. India had a mixed educational distribution—about a quarter of women had no education (27%) and more than 60% had secondary or higher education. In Nigeria, about three-quarters of women had secondary or higher education, while in Kenya two-fifths had only primary education and more than half have secondary or higher; these relatively high education levels reflect the focus on major urban areas in this analysis.

Across the countries, the wealth groups calculated for these urban areas were nearly evenly split; the wealth groups were created as quintiles, so it is expected that about 20% would be in each category.

Senegal had the highest percentage of Muslims (94%) of the 4 countries. In Nigeria, about half the population was Muslim, and the remaining half were Christian (Protestant or Catholic). In Kenya, the majority of the women were Protestant (64%); Catholics were the next most common religious group (22%), followed by Muslims (12%). In India, the majority of the women were Hindu (81%), and the remainder were predominately Muslim.

Finally, in Kenya and Nigeria (where all women were included in the samples), about a quarter were never married, and the remainder were predominately currently in union (63% in Kenya and 66% in Nigeria).

### Program Exposure Variables at Midterm

Table 4 presents the country-specific exposure variables at midterm. In each country, there are different levels of reported recall of exposure to each Urban RH Initiative activity.

**Table 4.** Percentage of Women^a^ Recalling Exposure to Specific Program Activities at Midterm, by Country

**Program Activity**	**India (UP)**	**Kenya**	**Nigeria**	**Senegal**
Exposure to CHW in the last 3 months	22.52			
Ever saw any UHI TV program^b^	41.52			
Ever heard any UHI radio program^b^	5.32			
Attended FP/Tupange meeting in the last year		11.49		
Saw Tupange leaflet in the last year		32.58		
Saw Tupange poster in the last year		43.16		
Saw Shujazz comic book in the last year		16.94		
Heard *Jongo Love* radio program in the last year		16.29		
Saw episode of *Matatu* TV program in the last year		22.33		
Heard or seen “NURHI” in the last year			23.01	
Ever heard of language-specific NURHI radio programs			28.96	
Heard NURHI phrases/slogans^c^ in the last year			30.91	
Ever listened to language-specific NURHI radio programs			56.55	
Seen NURHI puzzle logo in the last year			26.81	
Received info on FP/birth spacing at a community event^d^ in the last year			20.85	
Heard general FP messages on the radio in last 3 months			63.38	
Saw FP on TV in last 3 months (NURHI was only group with TV advertisements during project period)			59.29	
Heard at least 1 ISSU radio program in the last year				40.57
Saw at least 1 ISSU TV program in the last year				66.43
Participated in at least 1 ISSU community activity in the last year				22.31
Heard an FP radio advertisement in the last year				47.59
Heard religious leader speak favorably about FP in the last year				27.18

Abbreviations: CHW, community health worker; FP, family planning; ISSU, l'Initiative Sénégalaise de Santé Urbaine; NURHI, Nigerian Urban Reproductive Health Initiative; UHI, Urban Health Initiative; UP, Uttar Pradesh.

a The matched analysis sample comprised, in India, women in union and not sterilized at baseline; in Kenya and Nigeria, all women; and in Senegal, women in union at baseline.

b The midterm questionnaire for India asked specifically about 3 UHI spots: (1) *Sambhal lunga*, about a wife taking control and going to see a doctor and to use a contraceptive method; (2) *Munna*, in which a husband adopts male sterilization after talking to a doctor and has a happy married life afterwards; and (3) *Kishton Mein*, a story about a couple who adopts female sterilization at the time of delivery because they don't want any more children. Each was asked related to TV and radio exposure separately.

c Includes “Get it Together”; “Know, Talk, Go”; “No Dulling”; and attending a family planning meeting led by someone wearing a program t-shirt.

d Includes association meetings, naming and freedom ceremonies, graduation events, Christmas/Eid celebrations, and weddings.

In India, more than 40% of women recalled exposure to a UHI television program whereas 23% reported exposure to a community health worker (CHW) and only 5% to a UHI radio program.

In Kenya, the most common Tupange demand generation activity known by the women was the print media, comprising the Tupange leaflets (33%) and posters (43%). Among the remaining activities (including the comic book and radio and television programs), between 16%–22% of women reported exposure. Only 11% of women reported exposure to Tupange-led group/club/professional association meetings where family planning was discussed. Notably, in Kenya, the midterm questionnaire did not ask specific questions on exposure to community outreach workers; this oversight will be corrected at endline.

In Senegal, more than two-thirds of women recalled exposure to the ISSU television program and more than 40% recalled exposure to an ISSU radio program and a family planning radio advertisement. Close to a quarter of women recalled exposure to community activities (22%) or heard a religious leader speak favorably about family planning (27%).

Finally, in Nigeria, radio was a common source of general family planning information (64%), as well as language-specific NURHI radio programs (57%). Likewise, more than half of women reported at midterm that they recalled seeing family planning television advertisements. During the observation period (eg, 2010–2012), NURHI was the only group airing family planning messages on television. Among the other NURHI demand generation activities, between 20% and 30% of women were exposed to the logo, slogans, and community events.

### Contraceptive Use at Baseline and Midterm

Table 5 presents the percentage of women using a contraceptive method (no method use, traditional method use, and modern method use) at baseline and midterm in each study country. Because our focus in this analysis is contraceptive adoption by midterm, we are particularly interested in the changes between the two time periods.

**Table 5.** Contraceptive Method Use Among Surveyed Women^a^ at Baseline and Midterm, by Country

**Type of Method**	**India**		**Kenya**		**Nigeria**		**Senegal**	
	**Baseline^b^**	**Midterm**	**Baseline**	**Midterm**	**Baseline**	**Midterm**	**Baseline**	**Midterm**
No method	39.10	37.12	50.99	46.46	68.89	61.29	71.04	66.12
Traditional method	24.24	25.32	4.23	7.81	7.83	8.92	2.94	2.36
Modern method	36.66	37.56	44.78	45.73	23.28	29.79	26.02	31.52

a The matched analysis sample comprised, in India, women in union and not sterilized at baseline; in Kenya and Nigeria, all women; and in Senegal, women in union at baseline.

b In India, when considering all women in union (including those were who were sterilized at baseline) who were surveyed at midterm (N = 5,790), at baseline, 48.91% were using a modern method, 17.19% were using a traditional method, and 33.89% were not using a method.

In India, we include two baseline measures: the distribution of use at baseline among women in the matched baseline–midterm analysis sample comprising women who were in union and not sterilized at baseline (ie, women with whom the family planning program could have an impact) as well as the baseline distribution of use among *all* women in union surveyed at midterm (to provide an accurate portrayal of baseline use in these Indian cities since sterilization is the most commonly used method). At baseline, in the full midterm sample (that includes some women who were sterilized at baseline), the prevalence of modern method use was 49% (Table 5, footnote b). Among the remaining women, about a third were non-users and 17% traditional method users. In the analysis sample, the percentage using at baseline was lower at 37%, and the percentages of women who were non-users and traditional method users were higher (39% and 24%, respectively) (Table 5). By midterm, the percentage of the analysis sample that was using a modern method (38%) was similar to the percentage using at baseline.

For Kenya, like India, the aggregate percentage of women using modern contraception between baseline and midterm remained about the same at 45%–46% among women surveyed at baseline and midterm. Use of traditional methods increased slightly between baseline and midterm.

In Nigeria and Senegal, modern contraceptive use increased by about 6–7 percentage points between baseline and midterm (Nigeria, 23% to 30%; Senegal, 26% to 32%). Traditional method use changed little in these two countries.

Overall, the results of modern method use in Table 5 are difficult to interpret because some women are aging in the follow-up period and thus are becoming more (or less) likely to use a modern method; multivariate analyses are needed to control for these aging effects as well as other factors such as education and wealth.

### Multivariate Analyses: Is Program Exposure Associated With Modern Contraceptive Use?

The results of the multivariate random effects logistic regression models are presented in figures that show odds ratios (ORs) and their significance for the key program exposure variables by country. (Estimation results from corresponding fixed effects models were similar, indicating that endogeneity of the key exposure variables was likely not a problem and thus are not reported in favor of the more precise random effects estimates.) In all countries, the models controlled for age group, education, wealth, religion, and city of residence. In Senegal, the models also controlled for general family planning messages in the mass media but this was not found to be significant. In Kenya and Nigeria, marital status was included in the model since the analysis sample includes women who are in union as well as those who are not (never married, separated, or divorced). A small number of other country-specific control variables are also included in the models.

**Figure 1.**
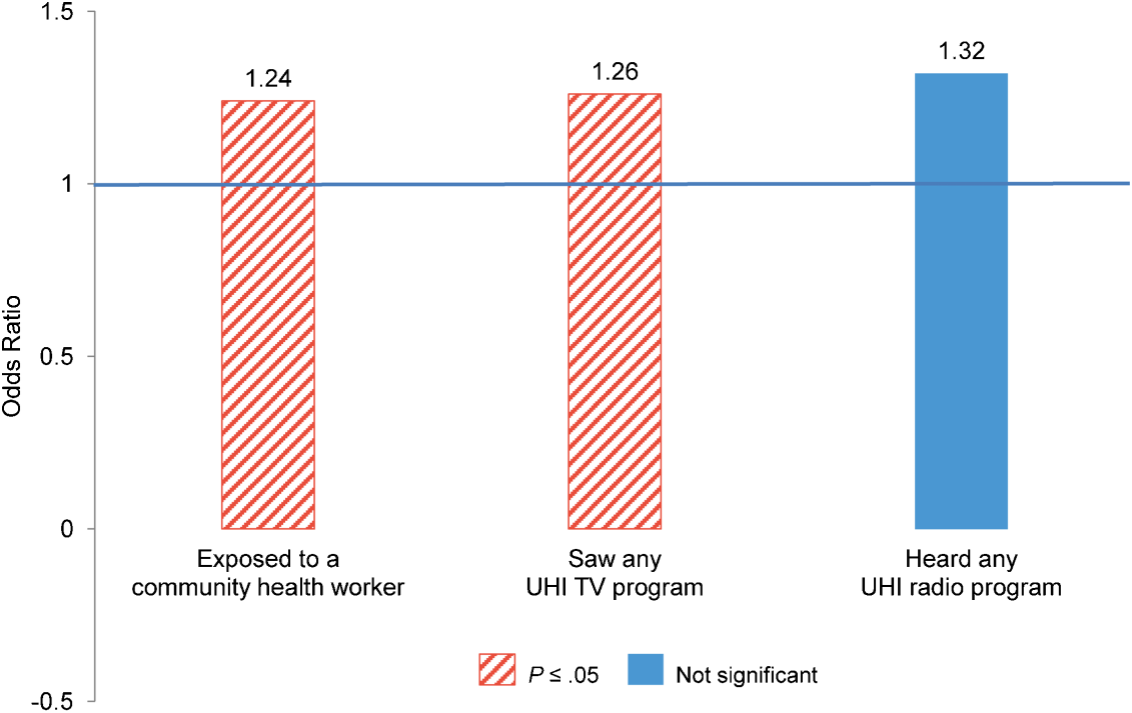
Odds Ratios From Random Effects Analysis of Demand Factors Associated With Modern Method Use Among Women in Union and Not Sterilized at Baseline in India Abbreviation: UHI, Urban Health Initiative. Model controls for age group, education, wealth, religion, city of residence, and other country-specific variables.

Figure 1 demonstrates that in India, women who recalled exposure to a CHW (OR = 1.24; *P* ≤ .05) and to any UHI television program (OR = 1.26; *P* ≤ .05) were significantly more likely to be modern method users at midterm than women who did not recall such exposure. While the odds ratio for UHI radio programs is large (OR = 1.32), it was not significant in the random effects models. This may be related to low overall recall of exposure to radio programs, as shown in Table 4.

In India, exposure to UHI television programs was significantly associated with modern method use.

**Figure 2.**
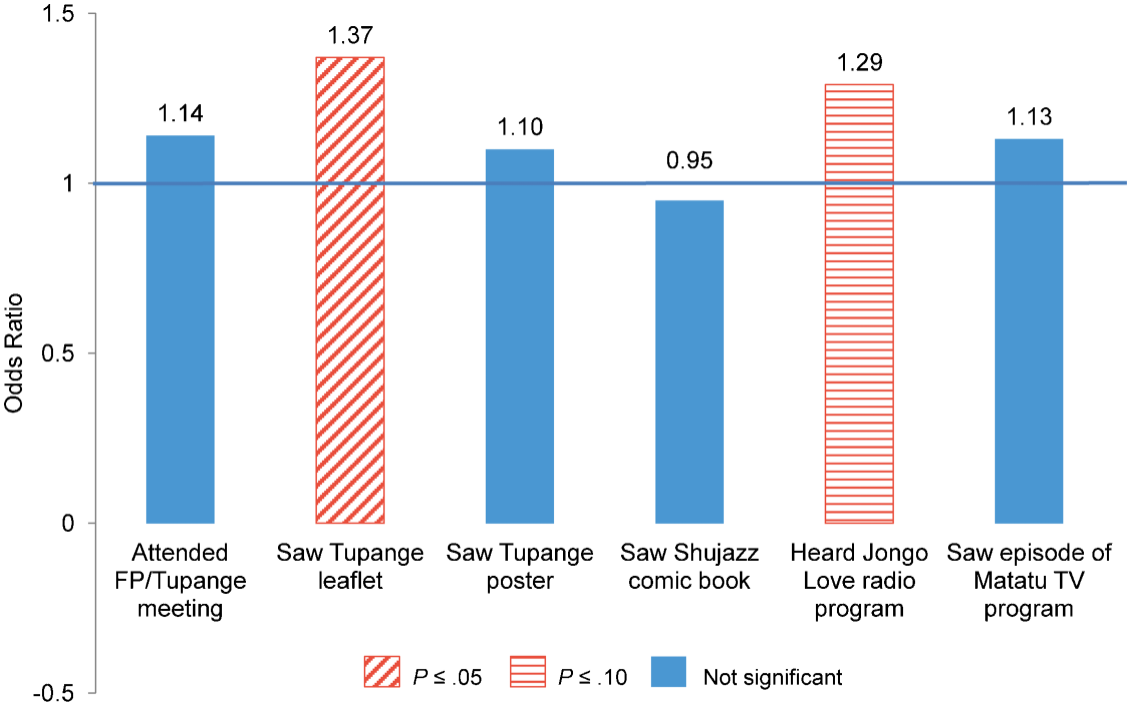
Odds Ratios From Random Effects Analysis of Demand Factors Associated With Modern Method Use Among Women in Kenya Abbreviation: FP, family planning. Model controls for age group, education, wealth, religion, city of residence, marital status, and other country-specific variables.

In Kenya, exposure to Tupange leaflets and brochures was significantly associated with modern contraceptive use between baseline and midterm (OR = 1.37; *P* ≤ .05) (Figure 2). In addition, exposure to Tupange's *Jongo Love* radio program was marginally significant (OR = 1.29; *P* ≤ .10).

**Figure 3.**
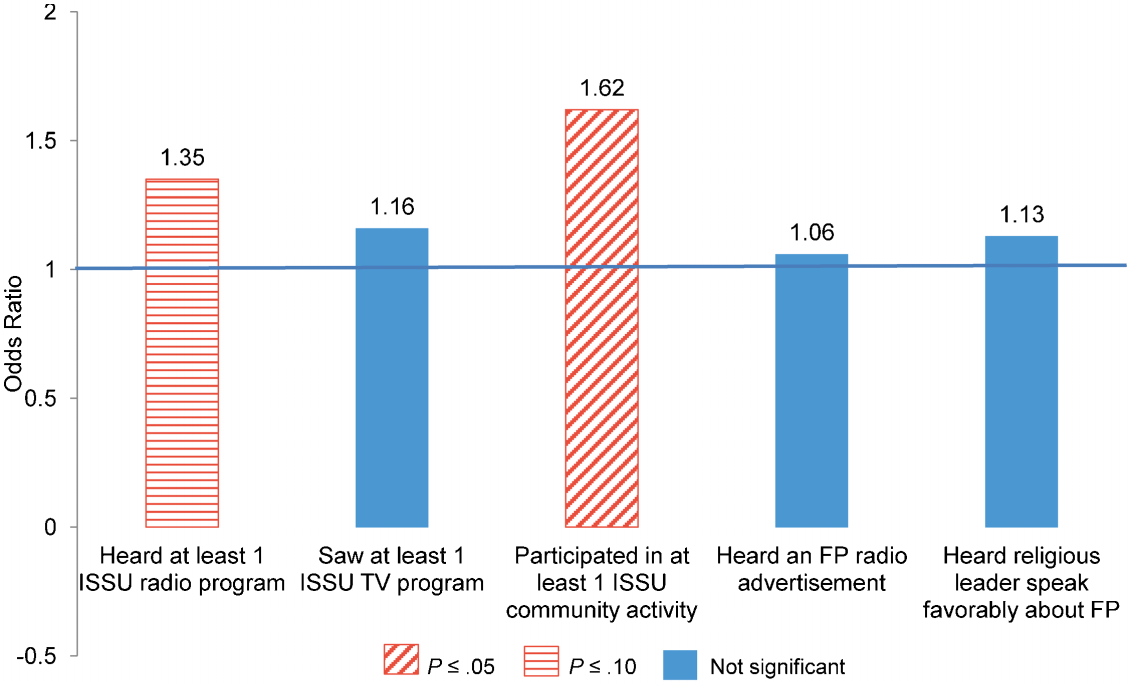
Odds Ratios From Random Effects Analysis of Demand Factors Associated With Modern Method Use Among Women in Union in Senegal Abbreviations: FP, family planning; ISSU, l'Initiative Sénégalaise de Santé Urbaine. Model controls for age group, education, wealth, religion, city of residence, and other country-specific variables.

In Senegal, women who reported participating in at least one ISSU-supported community activity, comprising a community conversation, a small group discussion, or outreach worker visits, were significantly more likely to be modern method users at midterm than women who did not report participating in any community activity (OR = 1.62; *P* ≤ .05) (Figure 3). This significance is notable given that it is the demand generation activity with the lowest reported exposure (22%). The other factor found to be marginally significant in Senegal is recalling hearing at least one ISSU-sponsored radio program in the last year (OR = 1.35; *P* ≤ .10). In these results, we did not find an effect of exposure to a religious leader speaking favorably about family planning on women's modern method use. However, in results not shown, when models were run using men's midterm data examining men's method use and attitudes toward family planning, we found that those men who recalled exposure to religious leaders speaking favorably about family planning were more likely to report family planning use as well as to have favorable family planning attitudes than men with no reported exposure to religious leaders (*P* ≤ .05).

**Figure 4.**
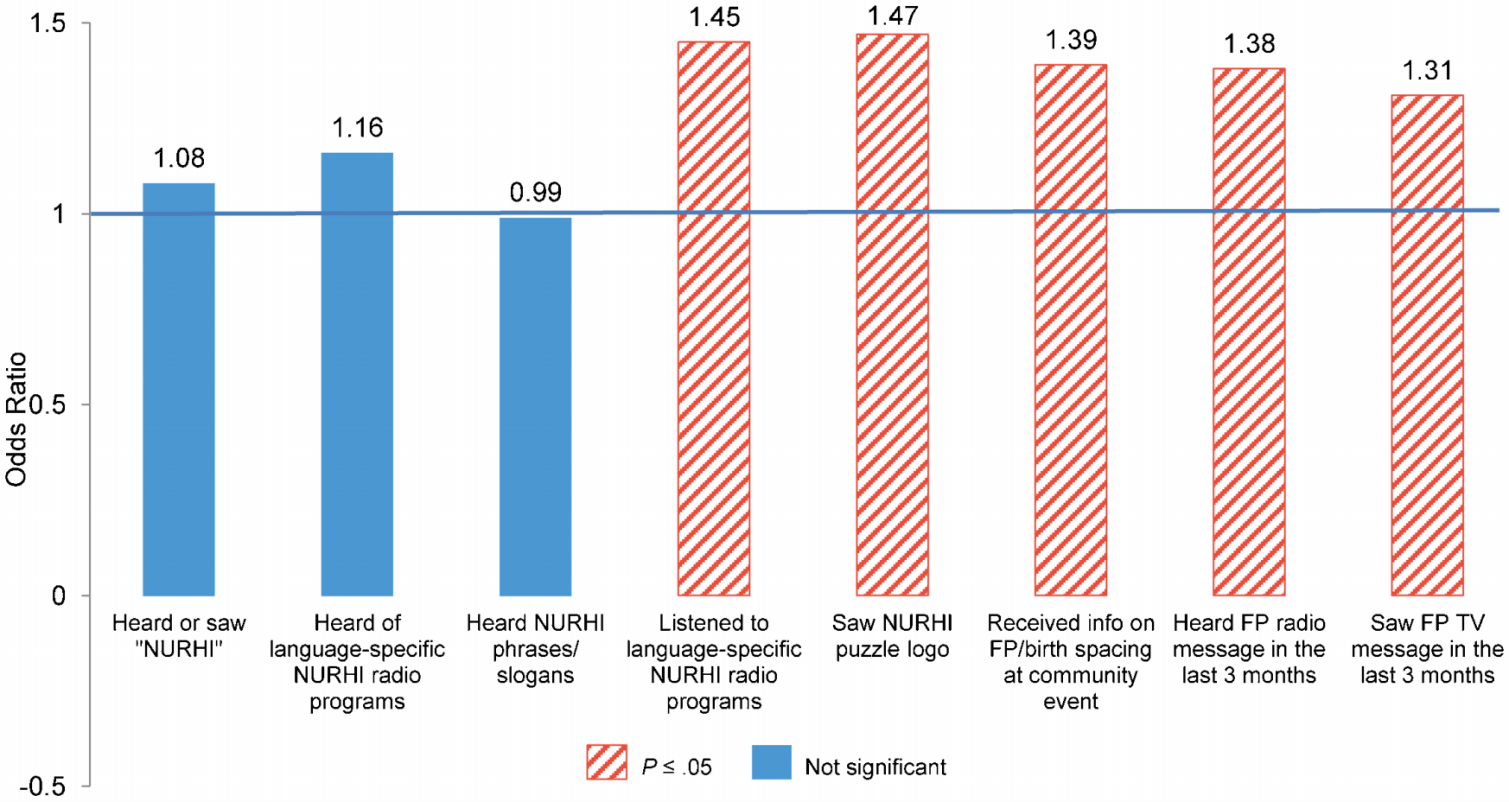
Odds Ratios From Random Effects Analysis of Demand Factors Associated With Modern Method Use Among Women in Nigeria Abbreviations: FP, family planning; NURHI, Nigerian Urban Reproductive Health Initiative. Model controls for age group, education, wealth, religion, city of residence, marital status, and other country-specific variables.

In Nigeria, women who recalled ever listening to local-language NURHI radio programs were significantly more likely to be modern method users at midterm than women who did not recall listening to these radio programs (OR = 1.45; *P* ≤ .05) (Figure 4). In addition, women who were familiar with the NURHI puzzle logo, a colorful logo that included the “Get It Together” slogan as part of the program branding, were also significantly more likely to be modern method users at midterm (OR = 1.47; *P* ≤ .05). The puzzle logo is used on all types of program materials, including on television, signs at health facilities, posters, and umbrellas. Women who recalled receiving information on family planning at any of the measured community events including association meetings, naming ceremonies, freedom ceremonies, graduation events, Christmas or Eid celebrations, and weddings, were significantly more likely to be modern method users at midterm than women who did not recall participating in these community events (OR = 1.39; *P* ≤ .05). Finally, women who recalled exposure to family planning messages on television in the last 3 months (considered to be NURHI television messages) were significantly more likely to be modern method users at midterm than women who did not recall exposure (OR = 1.31; *P* ≤ .05). A similar effect was found for exposure to general family planning messages on the radio (OR = 1.38; *P* ≤ .05), although this may have included non-NURHI radio activities as well. As noted earlier, the NURHI program has a strong focus on demand generation activities and theorizes that the multiple demand generation activities build upon one another. In results not shown, when women's recall of exposure to demand generation activities are summed, those women who reported exposure to more activities were significantly more likely to be users of modern contraception at midterm than those exposed to fewer activities. This supports NURHI's overall program theory of change. Similar results were found in analyses that summed up the key Tupange demand generation activities (also not shown).

**Figure:**
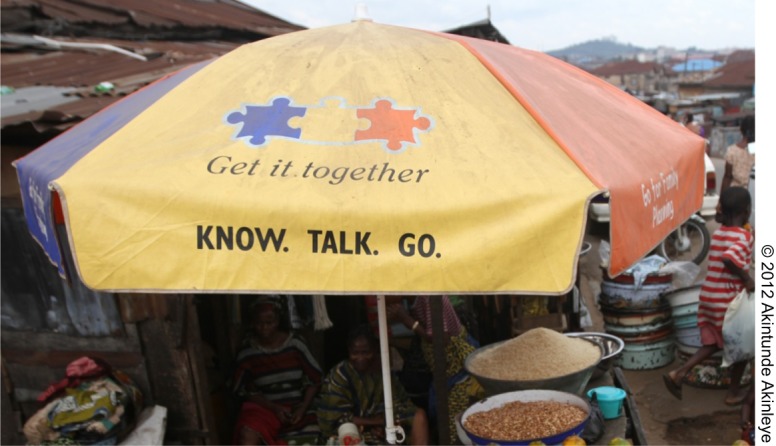
A roadside market umbrella in Mapo district in Ibadan, Nigeria, branded with the “Get it Together” NURHI puzzle logo, encourages people to “know” about family planning, “talk” with their partner about it, and “go” for family planning services.

## DISCUSSION

To date, there is little rigorous evaluation evidence of family planning programs targeting urban areas due to difficulties in identifying comparison groups. These findings, which use longitudinal data from women in 14 initial program implementation cities, are a first step in informing future family planning programs in urban settings. Of note are the different study contexts. India and Kenya started at higher levels of modern contraceptive use than Nigeria and Senegal, but the types of contraceptive methods used were different in the countries, particularly among the urban poor. Most poor users in India used female sterilization whereas most poor users in Kenya used injectables.^18^^–^^21^ In Nigeria and Senegal, family planning use is lower overall than in India and Kenya, with a common reliance on injectables in both countries as well as on condoms in Nigeria and on pills in Senegal.

Several interesting and informative results about demand generation activities emerge from our analysis. First, in each country where community outreach was measured, women who were exposed to community activities were significantly more likely than their counterparts who were not exposed to such activities to be modern method users at midterm. These findings are robust in the sense that models (ie, fixed effects) that explicitly correct for the potential endogeneity of program exposure (specifically, recall) offered similar results. In urban settings, it appears that interpersonal communication activities are important strategies for encouraging family planning use. Given that there continue to be a large percentage of women in urban settings that have myths and misconceptions about family planning,^18^^–^^20^ interactions with peers and health workers may be an influential strategy to change these problematic attitudes.

Second, local radio programs were also an effective means to encourage women to use family planning. Local radio programs were found to be significant in all 3 African countries but not in India, although the television programs in India that had the same themes (and greater exposure) as the radio programs were significant. Local radio programs were able to address specific concerns of urban, and particularly urban poor, populations as was done through Tupange's *Jongo Love* program in Kenya and through NURHI's radio magazine entertainment-education programs in Nigeria.

Television was also significant in India and Nigeria. In urban Nigeria, there is widespread access to television (about 89% of women in study cities watched television^19^) and thus can be an important approach to influence attitudes about and discussion of family planning topics in urban Nigerian settings.

In Kenya and Nigeria, specific program slogans and materials blanketed across the cities were found to be associated with increased modern method use. Program branding was less common in India and Senegal where the consortia worked to align their actions more closely with their respective government programs. These are strategic decisions that may be related to long-term program sustainability and that need to be made based on the country and urban context of future programs.

Notably, in Senegal, where religious leaders play an important role in daily life, we did not find an effect of exposure to religious leaders on women's contraceptive use. That said, in results not shown, recall of exposure to religious leaders was significantly related to men's reported approval and use of family planning. In a context such as Senegal, where more than 90% of the population is Muslim, it seems that working with influential religious leaders is an important strategy to influence contraceptive use directly among men and indirectly among women through their husbands.

Working with influential religious leaders may be an important way to influence contraceptive use directly among men and indirectly among women through their husbands.

Each Urban RH Initiative country program used the results of the midterm evaluation to make midcourse corrections. For example, UHI in India put greater emphasis on CHWs and community outreach activities in slum areas. Also, consortium-led television activities in Kenya and Senegal were dropped while radio programming in Nigeria and Senegal was continued or expanded. In addition to the fact that television programming is expensive, in Kenya and Senegal, reported exposure to television did not have an effect on contraceptive use. In Senegal, ISSU's television program was aired a small number of times on a limited number of television stations but on stations and times that had high coverage. The television programs did lead to high exposure, but they did not necessarily lead to discussion of family planning and to program impacts. On the other hand, the local radio programs that aired numerous times in Senegal were more effective, even with lower overall exposure. In Kenya, the main television program aired, called *Matatu Sema Kitu*, was an interactive show in matatu (taxis) that aimed to foster dialogue about family planning. While there appears to have been wide viewership estimated by the program, the activity did not have a significant effect on contraceptive use.

### Limitations

This analysis is not without limitations. First, because the midterm evaluation was seeking quick results for midcourse corrections, we did not obtain supply side information to determine whether short-term changes in quality and access to family planning may have influenced the results. Second, because we did not collect midterm data in the delayed intervention sites, we lack variation in the exposure variables that comes with comparison sites. Third, from the available data, it is not possible to know whether exposure preceded family planning use or vice versa; thus, the focus of this analysis is on associations rather than on causality. Fourth, the data do not permit an assessment of the *quality* of the CHW (or community event) interactions; this requires more in-depth, qualitative data. Fifth, the information on program exposure is based on respondent recall of family planning programs and messages. It is possible that other women were exposed but were already using family planning or, on the other hand, were exposed but not interested in family planning and thus did not report recalling the messages. Future analyses that examine women's (and men's) ability to recall key messages, intensity of exposure, and length of exposure would be informative for understanding the relationships between demand generation activities and modern method use. Finally, because smaller numbers of women were surveyed at midterm than initially intended, it was not possible to examine the full scope of transitions in modern contraceptive use (adoption, discontinuation, switching, and non-use). The 2014/15 endline evaluation will survey a larger sample and permit a more refined assessment of contraceptive use patterns over time as well as of the association of changes in family planning use with program activities.

## CONCLUSION

Targeted demand generation activities can make an important contribution to increasing modern contraceptive use in urban areas and could impact Millennium Development Goals for improved maternal and child health and access to reproductive health for all. These demand generation activities can be undertaken at multiple levels including community-level activities such as outreach by a health worker, a family planning worker, or a religious leader. Radio programs and, in particular, local radio programs that target key urban populations including younger and poorer groups are also likely to be successful at increasing modern method use.

## Notes

**Peer Reviewed**

**First published online:** 2014 Nov 6
